# Proteomic characterization of the TgF344 AD rat model of Alzheimer’s disease as a function of disease and therapeutic treatment

**DOI:** 10.1002/alz.090366

**Published:** 2025-01-03

**Authors:** Kalyani Chaubey, Jaymie Voorhees, Edwin Vázquez‐Rosa, Youngmin Yu, Xinming Wang, Sarah Barker, Kathryn Franke, Coral Cintrón‐Pérez, Noelle S. Williams, Bindu D Paul, Andrew A. Pieper

**Affiliations:** ^1^ Institute of Transformative Molecular Medicine, Case western Reserve University, Cleveland, OH USA; ^2^ University Hospitals Cleveland Medical Center, Cleveland, OH USA; ^3^ Case Western Reserve University, Cleveland, OH USA; ^4^ Louis Stokes Cleveland VA Medical Center, Cleveland, OH USA; ^5^ Inotiv, St. Louis, MO USA; ^6^ University of Toledo, Toledo, OH USA; ^7^ Department of Pathology, Case Western Reserve University, Cleveland, OH USA; ^8^ Department of Biochemistry, University of Texas Southwestern Medical Center, Dallas, TX USA; ^9^ Johns Hopkins University, Baltimore, MD USA; ^10^ Weill Cornell Medicine of Cornell University, New York, NY USA; ^11^ Department of Neurosciences, Case Western Reserve University, Cleveland, OH USA

## Abstract

**Background:**

Alzheimer’s disease (AD) is a severe neurodegenerative condition that affects millions of people worldwide. The TgF344 AD rat model, which exhibits early depression‐like behavior followed by later cognitive impairment, is widely used to evaluate putative biomarkers and potential treatments for AD. The P7C3 neuroprotective compounds have shown protective efficacy for both brain pathology and neuropsychiatric impairment in this model. To better understand the underlying pathophysiology and the mechanisms underlying therapeutic protection, we are investigating the associated changes in signaling pathways.

**Method:**

We conducted unbiased label‐free proteomic analysis of AD rat brains. Animals were treated daily for 6 months with either P7C3 compounds or vehicle, from 9 months of age to 15 months of age. At 15 months of age, cognitive function and depression‐like behavior were evaluated, and brain tissue was isolated for the proteomic study. Differentially expressed proteins in AD and AD animals treated with P7C3 compounds were categorized into functional categories and pathways to provide insight into disease pathology and treatment.

**Result:**

At this stage, rats had developed depression‐like behavior, but did not yet display cognitive impairment. We found 855 differentially expressed proteins in AD vehicle rats compared to WT littermate vehicle rats. Interestingly, 125 of these proteins returned to normal WT levels in AD animals treated with P7C3. Our study showed that major metabolic pathways and pathways related to neurodegeneration were highlighted at this time point. Most of these metabolic and neurodegenerative pathway‐related proteins returned to normal levels in TgF344 AD rats treated with P7C3 compounds.

**Conclusion:**

Pathway analysis of the differentially expressed proteins in AD that were normalized by P7C3 treatment were enriched in oxidative phosphorylation, glycolysis, tricarboxylic acid cycle, autophagy, tight junction, steroid and bile acid biosynthesis pathways. This provide insights into the pathophysiology of this AD model and complementary insights into future therapeutic opportunities.

